# Prevalence of hysterectomy and associated factors in Brazilian women aged 50 and older: findings from the Brazilian Longitudinal Study of Ageing (ELSI-Brazil)

**DOI:** 10.1186/s12889-024-19231-0

**Published:** 2024-06-30

**Authors:** Letícia Oliveira Afonso, Victória Wollf Beirith, Claudia Rosa de Andrade, Eliane Traebert, Cesar de Oliveira, Jefferson Traebert

**Affiliations:** 1 School of Medicine, Universidade do Sul de Santa Catarina, Avenida Pedra Branca, 25, Palhoça, SC 88132-260 Brazil; 2 Graduate Program in Health Sciences, Universidade do Sul de Santa Catarina, Avenida Pedra Branca, 25, Palhoça, SC 88132-260 Brazil; 3 Department of Epidemiology and Public Health, University College London, 1-19 Torrington Place, London, WC1E 6BT UK

**Keywords:** Hysterectomy, Gynaecologic surgical procedures, Epidemiology, ELSI-Brazil

## Abstract

**Background:**

Hysterectomy is a gynaecological surgical procedure in which the uterus is removed as a treatment for both malignant and benign gynaecological diseases. A hysterectomy is also performed to minimise risks in women with problems related to the uterus. This study aimed to estimate the prevalence of reported hysterectomy and associated risk factors in Brazilian women aged 50 and older.

**Methods:**

A cross-sectional study using data from the Brazilian Longitudinal Study of Ageing (ELSI-Brazil) was conducted. A total of 5,293 women aged 50 and over who participated in the ELSI-Brazil study in 2015 and 2016 were included. The prevalence rate of hysterectomy was estimated and the main reasons for performing the surgery were identified. The bivariate analyses utilised the chi-square test, while multivariate analyses employed Poisson regression with a robust estimator.

**Results:**

The reported prevalence of hysterectomy was 17.8%. The most prevalent reason for the surgery was the presence of uterine myoma. Significant and independent associations were observed in women aged 63 and older, married, having undergone preventive exams, hormonal treatment, had up to three deliveries and having a private health plan.

**Conclusion:**

The main objective of the study was achieved. The prevalence of hysterectomy in Brazilian women aged 50 and older was 17.8%. Significant associations were observed with participants’ sociodemographic and clinical characteristics reinforcing the importance of considering the reproductive characteristics of women as indicators of health status.

## Background

Hysterectomy is the second most prevalent surgery in women of reproductive age performed in Brazil’s Unified Health System (*Sistema Único de Saúde* - SUS), being surpassed only by caesarean section. [1]. In 2015, 53,753 hysterectomies were performed in the SUS with approximately 300,000 women indicated for this surgery [2, 3]. Therefore, the probability of women undergoing a hysterectomy during their lifetime ranges from 30 to 40%, as it is a resource for the treatment of gynaecological diseases, both malignant and benign [4].

Previous evidence [2, 3] has shown that sociodemographic characteristics, the long-term use of contraceptives and reproductive patterns may be contributing factors to the increase in hysterectomy rates. In India, for example, excessive menstrual bleeding followed by fibroids was the leading cause of hysterectomy. The main determinants were urban residency, working women and being from the most economically affluent group. Hysterectomy was associated with diabetes, hypertension, and joint diseases [5]. On the other hand, in Canada, hysterectomy rates were higher for the lowest neighbourhood educational quartile compared with the highest, higher with rural compared with urban dwelling, varied with local health service delivery area, and also varied non-linearly with neighbourhood income quintile [6].

In general, hysterectomy is performed to minimise the risk of life-threatening outcomes in women with problems related to the uterus and, consequently, allow them to enjoy a better quality of life [7]. Among the most frequent indications are uterine leiomyomas, pelvic organ prolapses, pelvic pain or infection (e.g., endometriosis, pelvic inflammatory disease), abnormal uterine bleeding, and malignant/premalignant tumours [8]. Although hysterectomy is the elective treatment for most gynaecological malignancies, the primary indication for this surgical procedure is benign gynaecological diseases [8].

The surgery can, however, impact women’s lives, either in terms of mental health, with the potential of triggering depressive symptoms, or in terms of self-image, as well as on their marital relationship and sexuality [9, 10]. Women undergoing total hysterectomy may have their sexual function impaired due to psychosocial and physical aspects associated with the surgical procedure [11]. Furthermore, the hysterectomy procedure has been associated with high rates of short- and long-term complications [12, 13]. In the long run, sexual dysfunction may occur, either by persistent or recurrent reduction in libido, arousal, and orgasm, along with the occurrence of pain, which can significantly limit women’s quality of life [13–15]. Gomes et al. have shown that hysterectomy was independently associated with frailty, reinforcing the importance of considering the reproductive characteristics of women as indicators of overall health status [16].

As to morbidity and mortality rates, they are higher in abdominal hysterectomies than in minimally invasive surgeries. Patients undergoing abdominal hysterectomy have a threefold increased risk of morbidity and mortality when compared to those undergoing laparoscopic hysterectomy [17, 18].

Hysterectomy is a procedure of extreme relevance in a woman’s life. Therefore, it is important to research hysterectomy to gain a better understanding of its prevalence and associated risk factors, particularly in women aged 50 and older. This will ultimately provide valuable evidence for shaping public health policies and reducing risk factors, morbidity, and mortality. Therefore, this study aimed to estimate, for the first time, the prevalence of hysterectomy and associated factors in Brazilian women aged 50 and older using a nationally representative sample of older Brazilian women.

## Methods

This was an epidemiological study with a cross-sectional design using data from the Brazilian Longitudinal Study of Ageing (*Estudo Longitudinal da Saúde dos Idosos Brasileiros -* ELSI-Brazil). ELSI data is publicly available at http://elsi.cpqrr.fiocruz.br/.

ELSI-Brazil aimed to assess the social and biological determinants of ageing and their consequences for the individual and society. The sample was designed to be representative of the community-dwelling Brazilians aged 50 or older. To ensure that the sample represented the urban and rural areas of the small, medium, and large municipalities, the ELSI-Brazil sampling used a design with selection stages, combining stratification of primary sampling units (municipalities), census tracts, and households. The municipalities were allocated to four strata depending on their population size: first stratum (≤ 26,700 inhabitants from 4,420 municipalities); second stratum (26,701 − 135,000 inhabitants from 951 municipalities); third stratum (135,001–750,000 inhabitants from 171 municipalities); and fourth stratum (> 750,000 inhabitants from 23 municipalities). All residents in the selected households aged 50 years or older were eligible for interview. We employed an inverse sampling design to mitigate nonresponse bias without enlarging the sample size. The final sample consisted of participants from 70 municipalities across major Brazilian regions. Sample weights were derived to account for differential probability of selection and differential nonresponse. More details on methodological aspects, including sampling can be found in Lima-Costa et al. [19].

Data was collected through interviews with women residents aged 50 or older. The inclusion criteria of the present study were data from women aged 50 years or over who participated in the ELSI-Brazil project in the years 2015 and 2016.

The dependent variable of this study was the report of performing a hysterectomy through the following question: “Did you have a hysterectomy, that is, was your uterus removed?” The answer possibilities were “yes, no, don’t know”. The reasons were asked as follows: “According to the doctor, what was the reason for removing the uterus?” The possible answers were uterine myoma, uterine prolapse, endometriosis, gynaecological cancer, pregnancy or childbirth complications, abnormal vaginal bleeding, other, don’t know/did not answer.

The covariates included were: age, dichotomised by the median of the sample distribution (63 years old); self-reported skin colour (dichotomised in white or non-white); education in years of study (dichotomised in up to 8 years of study, corresponding to the middle school; more than 8 years of study); marital status (with or without a partner); region of residence in large Brazilian geographic regions (North, Northeast, Midwest, Southeast and South); area of residence (urban and rural); current smoker (no/yes); if had already taken a preventive exam for cervical cancer (never did; already done); number of birth (up 3; more than 3); hormone treatment to relieve menopausal symptoms such as pills, patches, gels or injections (no/yes); visited the doctor in the last 12 months (no/yes) and having a private health plan (no/yes).

Data were entered into Excel spreadsheets and subsequently exported to SPSS Statistics for Windows, version 18.0 (SPSS Inc., Chicago, Ill., USA). Initially, a descriptive analysis of the variables was performed. Bivariate analysis between exposures and outcome (prevalence of reported hysterectomy) was performed using the chi-square test. All statistically significant variables at a *p* < 0.05 value and those with a *p* < 0.20 value were included in a multivariate analysis model using Poisson Regression analysis with a robust estimator. The prevalence ratios and their relevant 95% confidence intervals were estimated. Independently statistically significant associations at *p* < 0.05 were maintained in the final model.

The ELSI-Brazil research project was submitted to and approved by the Research Ethics Committee of the René Rachou Research Center/Oswaldo Cruz Foundation, Brazil under CAAE Number 34649814.3.0000.5091.

## Results

In this study, we included data from 5,293 women aged 50 or older who participated in the ELSI-Brazil study. The average age of the women was 64.2 years (SD = 10.2), 60.1% declared themselves as black, brown, or indigenous and 68.2% had up to 8 years of education. The prevalence of reported hysterectomy was 17.8% (95% CI 16.7; 18.8) i.e., totalling 940 women.

Table 1 summarises the reasons why the participants underwent a hysterectomy. Among those who were aware of the reasons, the most prevalent reason reported was the presence of uterine myoma (11.1%) and abnormal vaginal bleeding (1.8%). However, it was found that 82.6% of the women interviewed did not know the reasons that led them to undergo the hysterectomy procedure.

**Table 1:** Reasons for hysterectomy in women aged 50 and over. ELSI-Brazil, 2015–2016. (*n* = 940)

Reasons	Hysterectomy in women 50 years or older	
	**n**	%
Uterine myoma	104	11.1
Abnormal vaginal bleeding	17	1.8
Gynaecological cancer	6	0.7
Uterine prolapse	6	0.7
Complications of pregnancy or childbirth	5	0.5
Endometriosis	5	0.5
Other	21	2.2
Don’t know	776	82.6
Total	940	100.0

In the bivariate analysis, the results showed that women with more than 8 years of schooling (*p* = 0.018), in a stable relationship (*p* = 0.006), non-smokers (*p* = 0.027), who had already undergone preventive exams (*p* < 0.001), who had had up to three deliveries (*p* = 0.001), who had undergone hormonal treatment (*p* < 0.001), who had seen a doctor in the last 12 months (*p* = 0.004) and having a private health plan (*p* < 0.001) reported a higher occurrence of hysterectomy (Table 2).

**Table 2:** Results of the association study between the prevalence of hysterectomy in women aged 50 years and over and the independent variables studied. ELSI-Brazil, 2015–2016

Variables	Hysterectomy in women 50 years or older			
	Yes	No	Total	*p* value
	*n* (%)	*n* (%)	*n* (%)	
**Age years (median) (** ***n*** ** = 5,293)**				0.055
Up to 63	464 (16.8)	2,299 (83.2)	2,763 (52.2)	
Over 63	476 (18.8)	2,054 (81.2)	2,530 (47.8)	
**Skin colour (** ***n*** ** = 5,099)**				0.543
Not white	556 (18.2)	2,507 (81.8)	3,063 (60.1)	
White	356 (17.5)	1,680 (82.5)	2,036 (39.9)	
**Education (schooling years) (** ***n*** ** = 5,258)**				0.018
Up to 8	607 (16.9)	2,982 (83.1)	3,589 (68.2)	
Over 8	327 (19.6)	1,342 (80.4)	1,669 (31.8)	
**Marital status (** ***n*** ** = 5,293)**				0.006
No partner	470 (16.4)	2,393 (83.6)	2,863 (54.1)	
With partner	470 (19.3)	1,960 (80.7)	2,430 (45.9)	
**Region of residence (** ***n*** ** = 5,293)**				0.846
North	58 (15.6)	313 (84.4)	371 (7.0)	
Northeast	256 (18.0)	1,165 (82.0)	1,421 (26.8)	
Mideast	89 (17.6)	417 (82.4)	506 (9.5)	
Southeast	401 (17.8)	1,852 (82.2)	2,253 (42.7)	
South	136 (18.3)	606 (81.7)	742 (14.0)	
**Area of residence (** ***n*** ** = 5,293)**				0.226
Rural	129 (16.2)	665 (83.8)	794 (15.0)	
Urban	811 (18.0)	3,688 (82.0)	4,499 (85.0)	
**Current smoker (** ***n*** ** = 5,291)**				0.027
No	827 (18.2)	3,707 (81.8)	757 (14.3)	
Yes	113 (14.9)	644 (85.1)	4,534 (85.7)	
**Preventive exam (** ***n*** ** = 5,223)**				< 0.001
Never did	62 (10.2)	547 (89.9)	609 (11.7)	
Already done	865 (18.7)	3,749 (81.3)	4,614 (88.3)	
**Number of births (** ***n*** ** = 4,571)**				0.001
More than 3	367 (15.8)	1,954 (84.2)	2,321 (50.8)	
Up to 3	444 (19.7)	1,806 (80.3)	2,250 (49.2)	
**Hormone treatment (** ***n*** ** = 5,277)**				< 0.001
No	612 (15.7)	3,286 (84.3)	3,898 (73.9)	
Yes	325 (23.6)	1,0.054 (76.4)	1,379 (26.1)	
**Visited the doctor in the last 12 months (** ***n*** ** = 4,457)**				0.004
No	115 (14.8)	663 (85.2)	778 (17.5)	
Yes	706 (19.2)	2,973 (80.3)	3,679 (82.5)	
**Having a private health plan (** ***n*** ** = 5,290)**				< 0.001
No	645 (16.3)	3,306 (83.7)	3,951 (74.7)	
Yes	294 (22.0)	1,045 (78.0)	1,339 (25.3)	

The results of the multivariate analysis showed significant and independent associations between hysterectomy reports and age i.e., 63 years and older [PR = 1.02 (CI 95% 1.01; 1.03) *p* = 0.034], in a stable partnership [PR = 1.02 (95% CI 1.01; 1.03) *p* = 0.020], having undergone preventive exams [PR = 1.03 (95% CI 1.01; 1.05) *p* = 0.006], having had up to three deliveries [PR = 1.02 (95% CI 1.01; 1.03) *p* = 0.034], having undergone hormonal treatment [PR = 1.04 (95% CI 1.02; 1.06) *p* < 0.001] and having a private health plan [PR = 1.02 (95% CI 1.01; 1.03) *p* = 0.044] (Table 3).

**Table 3:** Results of the multivariate analysis between the prevalence of hysterectomy in women aged 50 years and over and the independent variables studied. ELSI-Brazil, 2015–2016

Variables	Histerectomy in women aged 50 or over			
	PR_c_ (CI 95%)	*p* value	PR_a_ (CI 95%)	*p* value
**Age years (median)**		0.055		0.034
Up to 63	1.00		1.00	
Over 63	1.01 (1.00; 1.02)		1.02 (1.01; 1.03)	
**Education (schooling years)**		0.018		
Up to 8	1.00		#	
Over 8	1.02 (1.01; 1.03)			
**Marital status**		0.006		0.020
No partner	1.00		1.00	
With partner	1.02 (1.01; 1.03)		1.02 (1.01; 1.03)	
**Current smoker**		0.027		
No	1.00		#	
Yes	1.02 (1.01; 1.03)			
**Preventive exam**		< 0.001		0.006
Never did	1.00		1.00	
Already done	1.05 (1.03; 1.06)		1.03 (1.01; 1.05)	
**Number of births**		0.001		0.034
More than 3	1.00		1.00	
Up to 3	1.02 (1.01; 1.03)		1.02 (1.01; 1.03)	
**Hormone treatment**		< 0.001		< 0.001
No	1.00		1.00	
Yes	1.05 (1.03; 1.06)		1.04 (1.02; 1.06)	
**Visited the doctor in the last 12 months**		0.004		
No	1.00		#	
Yes	1.02 (1.01; 1.04)			
**Having a private health plan**		< 0.001		0.044
No	1.00		1.00	
Yes	1.03 (1.02; 1.05)		1.02 (1.01; 1.03)	

PR_c_= crude prevalence ratio. PR_a_= adjusted prevalence ratio

## Discussion

This is the first study to investigate hysterectomy using a nationally representative sample of older Brazilian women. The prevalence of hysterectomy in women aged 50 or older found in the present study was 17.8%. Although there are no population-based studies in Brazil to observe the behaviour of the incidence rate, other studies point to a decrease in the incidence rates of hysterectomies. A recent study shows a decrease in hysterectomy incidence rates compared to the 20th century [20]. Another study also indicated a reduction in the prevalence of hysterectomy for benign situations [21]. Therefore, a careful analysis is necessary regarding the potential disadvantages of hysterectomy due to postoperative complications, difficult recovery and even a feeling of loss of femininity in some women [21].

The results of the present study indicate that the most common reason for hysterectomy was the presence of uterine fibroids. However, in a recent systematic review, endometrial cancer was reported as the main reason for hysterectomy [22]. It is important to note that this systematic review is based on clinical studies, while ours is based on community-based data. Therefore, caution should be exercised when making comparisons. Another study, carried out in Finland, showed changes in the reasons for performing hysterectomies. Until the 2000s, the main indication was uterine myoma, which has declined. Genital prolapses or incontinence is now the most prevalent cause [20].

It is also worth mentioning that most women in this study did not know the reason that led them to perform a hysterectomy. It is important to consider that the illiteracy rate among older Brazilian adults may be related to this result. According to data from 2022, an illiteracy rate of 16.3% was observed among women at a national level, reaching more than 30% in some regions of the country [23]. This fact highlights the importance of increasing self-knowledge about health and understanding comorbidities. Doctors need to explain diagnoses and treatments clearly to demystify pathologies and help patients understand the reasons for each treatment.

The results of the multivariate analysis showed that older women had a significant prevalence of hysterectomy greater than 2%. In the present study, age was dichotomised by the median of the age distribution i.e., 63 years. Women in the older age group had a statistically higher prevalence. However, there is no information on when the surgery was performed. The age at which hysterectomies are performed is an important variable and varies among regions and even over time. In Finland, the average age of all hysterectomies performed from 2014 to 2017 was 55 years [20].

There was a significant association between undergoing a hysterectomy and receiving preventive exams at least once in a lifetime. It is important to highlight that the Brazilian Unified Health System is public, universal, and free, complemented by private health services. However, providing equal access to the entire population remains a significant challenge. There are still extremely vulnerable segments of the population who are unable to access the healthcare system for a preventive gynaecological examination or even a medical consultation, which can increase the risk of gynaecological pathologies that could require treatment or, ultimately, a hysterectomy. It is hypothesised that women who seek healthcare may receive better diagnoses and undergo more surgical procedures. However, in recent years, some studies have shown that the prevalence of hysterectomies has decreased, especially in women of reproductive age. This is because the introduction of new less invasive treatments for uterine diseases has helped with the reduction in hysterectomies. Some examples are hormonal treatments, endometrial or uterine artery ablation, hysteroscopy, and other pharmacological treatments [24, 25].

Another significant association observed was between hormone treatment and hysterectomy. It is important to remember that the study design is cross-sectional, allowing only associations between the studied variables. There is no information available on whether hormone treatment was started before the hysterectomy. The possibility of the treatment being started after an operation can be considered high, as an outcome of the hysterectomy. A longitudinal design would be more appropriate to study the determinants of hysterectomy. The same reasoning should be considered regarding the variable visited doctor in the past 12 months. There is also a high possibility of women visiting health care providers for post-operative care or managing post-operative health. As highlighted by Paiva et al. [26], the preferred primary form of treatment recommended for leiomyomas and their consequences, such as abnormal uterine bleeding, before performing a hysterectomy is the use of hormones. Therefore, a possible explanation for the association found may be that before deciding on hysterectomy as the treatment of leiomyomas (one of the main causes of hysterectomy), and of other uterine pathologies, a first conservative approach using hormone treatment is indicated.

Our findings showed that women who had a private health plan had a 2% higher prevalence of hysterectomy. It is important to highlight the design of our study only allows us to raise hypotheses. A plausible explanation is that easier access to health procedures by those with private health plans may be linked to the higher prevalence of hysterectomies. Also, it could be related to the possible financial advantage of surgery brought to physicians through health plans [27–29]. Therefore, women who depend only on the public health system end up having unequal access to clinical investigation, diagnostic and therapeutic procedures, such as surgery [2, 27]. According to studies carried out in other countries and considering the specific characteristics of each region regarding the functioning of health plans, there could be a financial interest of doctors and private health plans in referring patients for hysterectomy, even when this procedure is not indicated and unnecessary [28, 29].

The significant association between hysterectomy and marital status has also been demonstrated in previous studies [7, 30]. Although the reason for this association is still not well established, it can be partially attributed to the fact that these patients have an active sex life, which puts them at greater risk of developing inflammatory processes or sexually transmitted diseases [7, 30]. On the other hand, a study [24] showed that married patients had a lower risk of requiring a hysterectomy. The authors suggested that this may be because women in a stable relationship are more likely to take greater care of their sexual and gynaecological health [27].

Another significant association found was between hysterectomy and parity, especially in those women with up to three children. However, parity as a risk factor for hysterectomy is controversial in the literature, as it correlates the surgical procedure with both nulliparity and a greater number of children [2]. Nevertheless, several studies have shown a positive association between hysterectomy and greater parity [7, 30, 31]. One hypothesis lies in the fact that oestrogen, present in pregnancy, is also the hormone that stimulates fibroid growth [32, 33]. Thus, when pregnant, a woman is exposed to a greater risk of requiring a postpartum hysterectomy, since there is a greater serum oestrogen circulating during this period, which may lead to increased growth of fibroids [34].

In the present study, educational level, a variable that usually influences hysterectomies, has lost its significance in the multivariate analysis. In the literature, it is well established that women with higher education have a lower risk of undergoing hysterectomy [7, 29–31]. This is because they have greater access to information about their gynaecological health and can communicate better with their doctors. Thus, they are more likely to undergo alternative treatments to hysterectomy [29, 30].

Another variable that lost its association with hysterectomy was current smoking. In this study, the bivariate analysis showed a significant association with non-smokers. However, it lost its significance in the multivariate analysis. A similar finding was reported by Piotto et al. [27] Therefore, further studies should be performed to elucidate the role of smoking in hysterectomy.

Some limitations impose caution when interpreting the results of our study. It included women aged 50 and over, an age at which women are near or are already in menopause. There is also no information on whether menopause would result from a natural physiological phenomenon or possibly artificial menopause, for example, by a bilateral oophorectomy. The information about menopause could be relevant, as it may be linked to hormonal treatment and parity. Another important limitation is the lack of information about the age at which the surgery was performed. The large number of women who did not know the reasons underlying their hysterectomy, preventing a more careful analysis of the reasons for the surgery is also a limitation. One cannot dismiss the possibility that the question regarding the topic required further explanation to reduce the high rate of negative responses, without unduly influencing the results. Memory bias must also be considered. Our results should be interpreted cautiously, as uterine myoma may not be the primary cause. Thus, increasing awareness among women about their gynaecological health and the necessary therapeutic procedures is crucial. Therefore, due to the importance of the topic, further studies are essential to address multifactorial issues, risks, and protective factors. This will contribute to a better understanding of women’s health in later life.

We can conclude that the main objective of the study has been achieved. Specifically, we estimated that 17.8% of women aged 50 or over in Brazil underwent hysterectomy. This estimation is based on a population-based and nationally representative sample, which provides data that were lacking in the literature. Associated factors included age, marital status, preventive examination, number of deliveries, hormone treatment and having a health plan. New studies are recommended to monitor the behaviour of rates, reinforcing the importance of considering the reproductive characteristics of women as indicators of health status.

## Data Availability

The datasets generated and/or analyzed during the current study are available in http://elsi.cpqrr.fiocruz.br/.
